# SSTDP: Supervised Spike Timing Dependent Plasticity for Efficient Spiking Neural Network Training

**DOI:** 10.3389/fnins.2021.756876

**Published:** 2021-11-04

**Authors:** Fangxin Liu, Wenbo Zhao, Yongbiao Chen, Zongwu Wang, Tao Yang, Li Jiang

**Affiliations:** ^1^School of Electronic Information and Electrical Engineering, Shanghai Jiao Tong University, Shanghai, China; ^2^Shanghai Qi Zhi Institute, Shanghai, China; ^3^School of Engineering and Applied Science, Columbia Univeristy, New York, NY, United States; ^4^MoE Key Lab of Artificial Intelligence, AI Institute, Shanghai Jiao Tong University, Shanghai, China

**Keywords:** spiking neural network, gradient descent backpropagation, neuromorphic computing, spike-time-dependent plasticity, deep learning, efficient training

## Abstract

Spiking Neural Networks (SNNs) are a pathway that could potentially empower low-power event-driven neuromorphic hardware due to their spatio-temporal information processing capability and high biological plausibility. Although SNNs are currently more efficient than artificial neural networks (ANNs), they are not as accurate as ANNs. Error backpropagation is the most common method for directly training neural networks, promoting the prosperity of ANNs in various deep learning fields. However, since the signals transmitted in the SNN are non-differentiable discrete binary spike events, the activation function in the form of spikes presents difficulties for the gradient-based optimization algorithms to be directly applied in SNNs, leading to a performance gap (i.e., accuracy and latency) between SNNs and ANNs. This paper introduces a new learning algorithm, called SSTDP, which bridges the gap between backpropagation (BP)-based learning and spike-time-dependent plasticity (STDP)-based learning to train SNNs efficiently. The scheme incorporates the global optimization process from BP and the efficient weight update derived from STDP. It not only avoids the non-differentiable derivation in the BP process but also utilizes the local feature extraction property of STDP. Consequently, our method can lower the possibility of vanishing spikes in BP training and reduce the number of time steps to reduce network latency. In SSTDP, we employ temporal-based coding and use Integrate-and-Fire (IF) neuron as the neuron model to provide considerable computational benefits. Our experiments show the effectiveness of the proposed SSTDP learning algorithm on the SNN by achieving the best classification accuracy 99.3% on the Caltech 101 dataset, 98.1% on the MNIST dataset, and 91.3% on the CIFAR-10 dataset compared to other SNNs trained with other learning methods. It also surpasses the best inference accuracy of the directly trained SNN with 25~32× less inference latency. Moreover, we analyze event-based computations to demonstrate the efficacy of the SNN for inference operation in the spiking domain, and SSTDP methods can achieve 1.3~37.7× fewer addition operations per inference. The code is available at: https://github.com/MXHX7199/SNN-SSTDP.

## 1. Introduction

Deep neural networks have made tremendous progress and become a prevalent tool for performing various cognitive tasks such as object recognition (Simonyan and Zisserman, 2015; Sandler et al., 2018), natural language processing (Devlin et al., 2018; Radford et al., 2019), and self-driving (Nedevschi et al., 2012; Liu et al., 2017), etc. To leverage the capability of deep neural networks in ubiquitous environments requires deployment not only on large-scale computers but also on portable edge devices (Han and Roy, 2020; Deng et al., 2021). However, the increasing complexity of deep neural networks, coupled with data flooding with distributed sensors continuously generates real-time content and places tremendous energy demands on current computing platforms. Spiking Neural Networks (SNNs) are often regarded as third-generation brain-inspired neural networks, and represent one of the leading candidates for overcoming computational constraints and efficiently exploiting deep learning algorithms in real (or mobile) applications whilst also being highly power-efficient (Deng et al., 2020; Rathi et al., 2020; Taherkhani et al., 2020).

SNNs consist of spiking neurons that transmit information in the form of electric event spikes via plastic synapses (Taherkhani et al., 2020). Event-driven computing capability is the fundamental characteristic of SNNs, supporting sparse and irregular input spike train, thereby reducing latency and power consumption of computation and communication (Lee et al., 2018). With the development of neuromorphic hardware supporting the SNN, such as Intel Loihi (Davies et al., 2018) and IBM TrueNorth (Akopyan et al., 2015), SNNs have gained increasing attention in both academia and industry. To date, shallow SNN structures (i.e., two fully connected layers) have been widely used for classification. However, training high-performance SNNs with competitive classification accuracy and less latency is a nontrivial problem, limiting their scalability in complex applications (Benjamin et al., 2014; Roy et al., 2019; Sengupta et al., 2019; Comsa et al., 2020; Han et al., 2020; Deng et al., 2021).

The existing training strategy for SNNs can be broadly divided into two categories, unsupervised learning and supervised learning (Roy et al., 2019). Unsupervised learning discovers the underlying features and structure of input data without using the corresponding labels. Spike-time-dependent plasticity (STDP) is a bio-plausible unsupervised learning mechanism that exploits the temporal difference between pre-and post-synaptic neuronal spikes to modulate the weights of neural synapses instantaneously (Pfister and Gerstner, 2006; Diehl and Cook, 2015; Bellec et al., 2018). It is a simple and fast training method that reflects the temporal correlations of pre-and post-synaptic spikes between neighboring (local) layers. However, the classification accuracy of SNNs trained based on the unsupervised learning represented by STDP is still lower than the results presented by state-of-the-art Artificial Neural Networks (ANNs). When it comes to supervised learning, it extracts internal features and structure given the training examples and target labels. The standard backpropagation (BP) is normally used for achieving state-of-art classification performance in ANNs by updating the network parameters to minimize the final output error of the network (He et al., 2016). The corresponding loss function is defined as the difference between the predicted output of the network and the expected target output (label). Meanwhile, the SNNs trained by supervised learning can achieve much better performance than the unsupervised ones, triggering recent works to use the BP-based learning algorithm to train SNNs by input binary spike events. However, training such SNNs is quite difficult. Since the spiking neurons communicate through discrete, non-differentiable spike events, which is fundamentally different from the continuous activations of non-spiking neurons such as the ReLU function in ANNs, it is impossible to transfer the BP-based learning mechanism to SNNs directly (Wu et al., 2021).

There have been some successful attempts to introduce the BP-based learning mechanisms into SNNs (Lee et al., 2016; Tavanaei et al., 2019; Zhou et al., 2019; Kheradpisheh et al., 2020; Fang et al., 2021; Mirsadeghi et al., 2021). The first approach is the spike-based BP, which treats the membrane potentials as differentiable activations of spiking neurons and trains the synaptics of SNNs in a layer-wise fashion (Tavanaei et al., 2019). The second approach is to use spike rates (frequency) to substitute the non-differentiable spike events (Liu et al., 2015). Although these two types of methods are suitable for gradient descent learning, it requires complicated procedures for computing the derivative of the loss function in spatial and temporal domains. The third approach is approximate methods, which estimate the surrogate gradient of the spike generation function (Mirsadeghi et al., 2021). However, this kind of approach incurs the strong assumption in backpropagating the error through the network using the chain rule. For instance, recent work proposes S4NN (Kheradpisheh et al., 2020), where they use a temporal version of the traditional BP-based learning to train a multi-layer SNN consisting of IF neurons. This method approximates the derivative of time with respect to the potential as −1 in the backpropagation process. Meanwhile, none of these methods take into account the temporal dynamics between pre-and post-synaptic spike timings and are efficient for hardware implementation with rate coding.

It is unclear which learning algorithm (i.e., unsupervised learning algorithm or supervised learning algorithm) is suitable for training the SNN (Roy et al., 2019; Deng et al., 2020, 2021; Lobo et al., 2020). Both STDP and spike-based BP learning have been demonstrated they can effectively capture the hierarchical features in SNN. On the one hand, the spiking neural networks trained solely on STDP-based methods lack competitive classification performance. On the other, BP-based SNN training methods usually lead to unstable convergence, and a slight variance on the hyper-parameters will have a great impact on the result. For these reasons, this study proposes utilizing STDP-based unsupervised learning to encourage the hidden layer to discover the local features and structures of the input patterns. In combination with the gradient-based supervised algorithm, it will guide the optimization in a global manner. The multi-layer spiking neural network consists of convolutional layers and pooling layers, followed by successive fully connected layers. Bio-plausible integrate-and-fire spiking neurons populate the layers in the SNN to process sparse spike trains that encode pixel intensities as the precise timing of spikes (temporal coding).

The first main contribution of this work is that it uses a time-based supervised learning method that employs the weight update mechanism derived from STDP to bypass the non-differentiable nature of the spike generation function in the BP process. In addition, we efficiently construct SNN architectures for different tasks, such as convolutional SNN for the large dataset (i.e., CIFAR-10), fully-connected SNN for the small dataset (i.e., Caltech 101 and MNIST). Next, we demonstrate the effectiveness of this methodology for visual recognition tasks on standard datasets (Caltech 101, MNIST, CIFAR-10). Finally, this study quantifies and analyzes the advantages of the proposed learning method compared to prior techniques in terms of latency and energy consumption. To the best of our knowledge, this work achieves the best performance SNN with the shortest latency (i.e., the number of time steps) in Caltech 101, MNIST, and CIFAR-10 datasets, among other learning methods.

Section 2 reviews related works and introduces the motivation of our work. Section 3 elaborates the proposed SSTDP learning algorithm. Section 4 then presents the experimental results, including experimental setups and evaluation metrics. It also discusses the comparison results with the recent works in terms of network performance, latency, and energy efficiency. Section 5 concludes the paper.

## 2. Related Work

### 2.1. STDP Methods

The STDP-based learning algorithm is a bio-plausible learning mechanism for SNNs. It is a promising approach that could improve the information processing capability of neurons by specifying different synapses for various types of input data and providing dynamic control over plasticity (Ferré et al., 2018; Kheradpisheh et al., 2018; Taherkhani et al., 2020).

**Figure 1:**
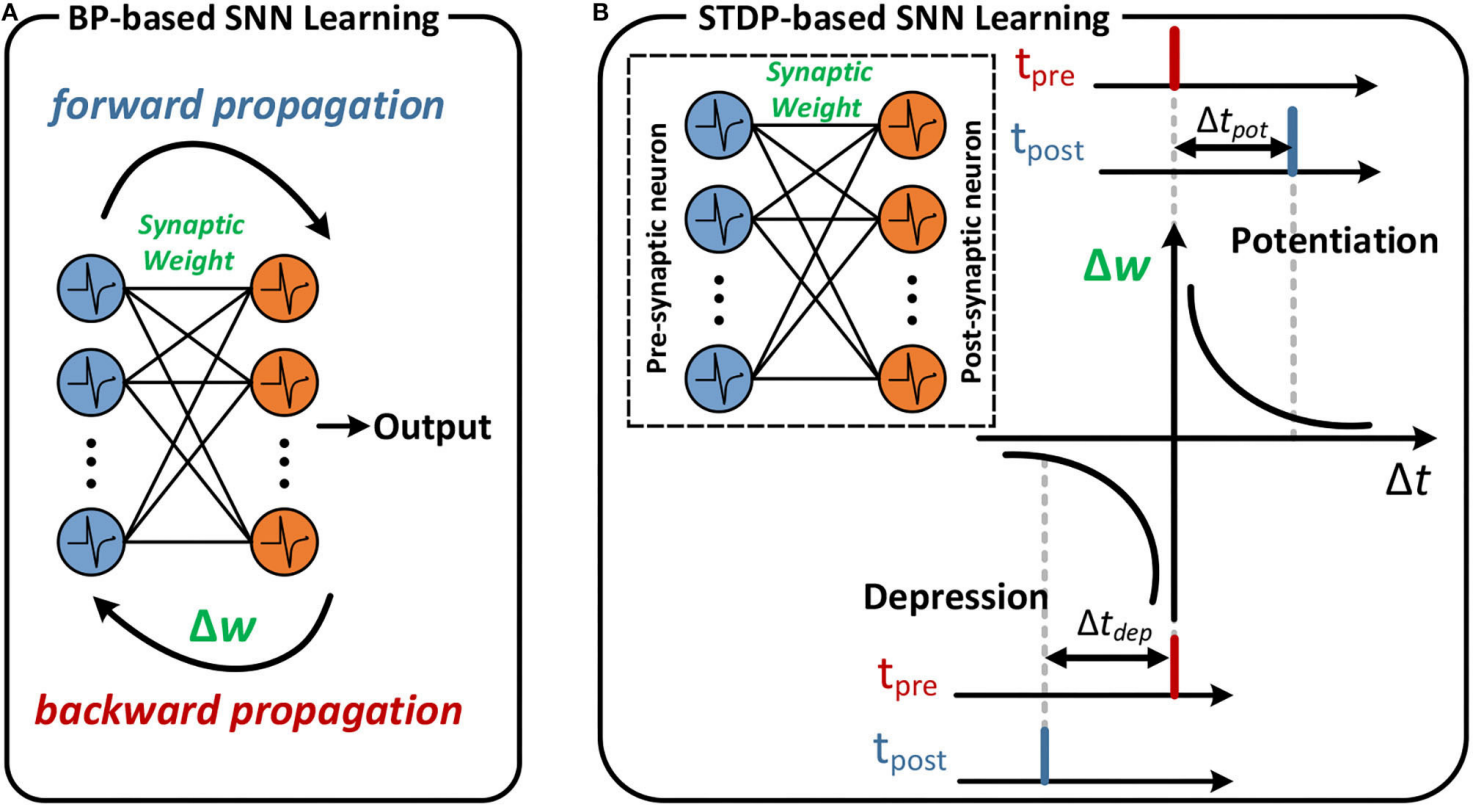
An example of BP-based learning and STDP-based learning. **(A)** Forward and backward propagation of the SNN. **(B)** If the post-synaptic neuron fires after the pre-synaptic spike arrives, the synaptic weight between pre- and post-synaptic neuron increases. The magnitude of change increases in proportion to Δ*t*_*pot*_. The reverse order leads to a decrease in synaptic weight in proportion to Δ*t*_*dep*_.

As shown in Figure 1B, the STDP-based learning algorithm is based on the temporal correlation (Δ*t* = *t*_*post*_ − *t*_*pre*_) between spike-time *t*_*pre*_ of the pre-synaptic neuron and spike-time *t*_*post*_ of the post-synaptic neuron to adjust the synapse weight as described in previous research tasks. Specifically, if the spike arrives at the pre-synaptic neuron *t*_*pre*_ earlier than the post-synaptic neuron fires the spike *t*_*post*_ within a given time window, the synapse weight is increased, which is called synaptic potentiation. The synaptic depression behavior is similar to potentiation. If the post-synaptic neuron fires the spike *t*_*post*_ later than the spike arrives at the pre-synaptic neuron *t*_*pre*_, the synapse weight is reduced and is referred to as a synaptic depression.

The STDP-based unsupervised feature learning using convolution-over-time in SNNs is proposed to encode representative input features (Srinivasan et al., 2018). The triplet STDP uses local variables called traces, as proposed in Pfister and Gerstner (2006). The traces associated with pre-synaptic neurons and post-synaptic neurons corresponding to two traces with fast and slow dynamics, respectively, to better extract the spiking dynamic features. A notable semi-supervised learning method based on STDP is outlined in the work of Lee et al. (2018), which uses STDP-based unsupervised learning to better initialize the parameters in pre-trained SNN and follows gradient-based supervised optimization. Tavanaei et al. (2019) proposed a learning rule that updates the synaptic weights using a teacher signal to switch between STDP and anti-STDP. However, it updates weights that only use local update rules and do not involve the gradient update mechanism of STDP. For all the methods mentioned above, all of which are based on STDP, the classification accuracy obtained from training is still lower than state-of-the-art results. Meanwhile, they all use rate-based coding to encode the information (i.e., multi-spikes in the spike train) and do not deal with time-based information directly (i.e., a single spike).

### 2.2. BP Methods

As illustrated in Figure 1A, the backpropagation algorithm is one successful method for training deep SNNs (Deng et al., 2020; Taherkhani et al., 2020). Although the spike-based BP algorithm can achieve better accuracy than the STDP-based learning algorithm (Kheradpisheh et al., 2020; Rathi et al., 2020; Mirsadeghi et al., 2021), it suffers from the same fundamental disadvantage: the computation of neurons theoretically occurs at the spike neuron and requires massive data and effort. Therefore, exploring the BP algorithm for temporal encoding is more efficient for hardware implementation. Meanwhile, considering that existing neuromorphic systems are time-driven execution mechanisms, for such systems, the computation of neurons occurs at each time step, and reducing the number of time steps while improving accuracy, should also be considered.

## 3. Approach

This paper proposes a novel learning method for the SNN with an accurate gradient descent mechanism and an efficient temporal local update mechanism by incorporating BP and STDP training methods. Thus, our method can effectively balance global and local information during training and can address some open questions regarding accurate and efficient computations.

### 3.1. Spiking Neural Network Components

#### 3.1.1. Network Architecture

**Figure 2:**
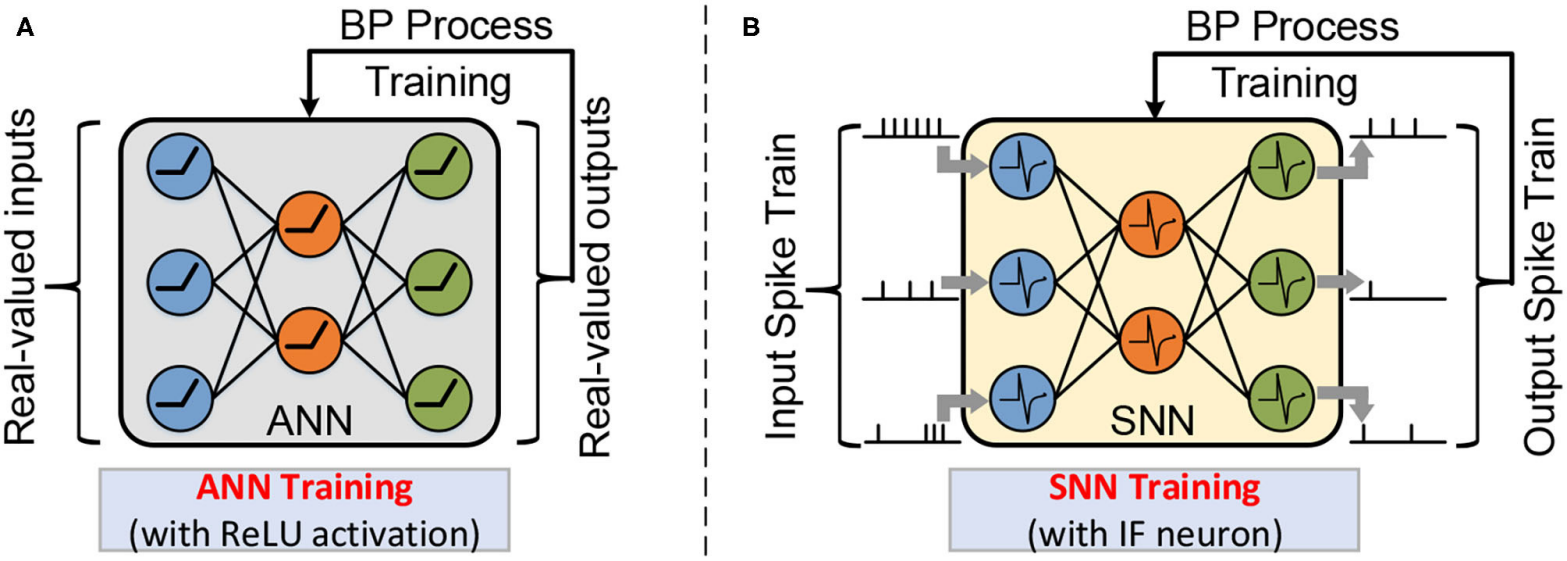
A multi-layer neural network is composed of an input layer, one or more hidden layers, and an output layer. The workload of natural ANN training with real-valued activation **(A)**; and SNN training with spatio-temporal spike trains **(B)**.

In Figure 2 describes the typical network architecture of ANN and SNN, which was used for the classification task. In the first layer, inputs that feed to the neuron are pixels of the input image in the ANN, while in the SNN, these pixels are converted into spike trains. In the hidden layer, non-spiking neurons in the ANN perform Multiply Accumulate (MAC) operations and then pass the result through the activation function (e.g., ReLU function) to generate the input for the next layer. In contrast, in SNN, each spiking neuron integrates weighted spikes and fires the output spike when the membrane potential exceeds the threshold potential. In the final output layer, each category corresponds to one neuron. The loss function of the output layer is defined as the difference between the predicted value and the expected value.

#### 3.1.2. Information Encoding

**Figure 3:**
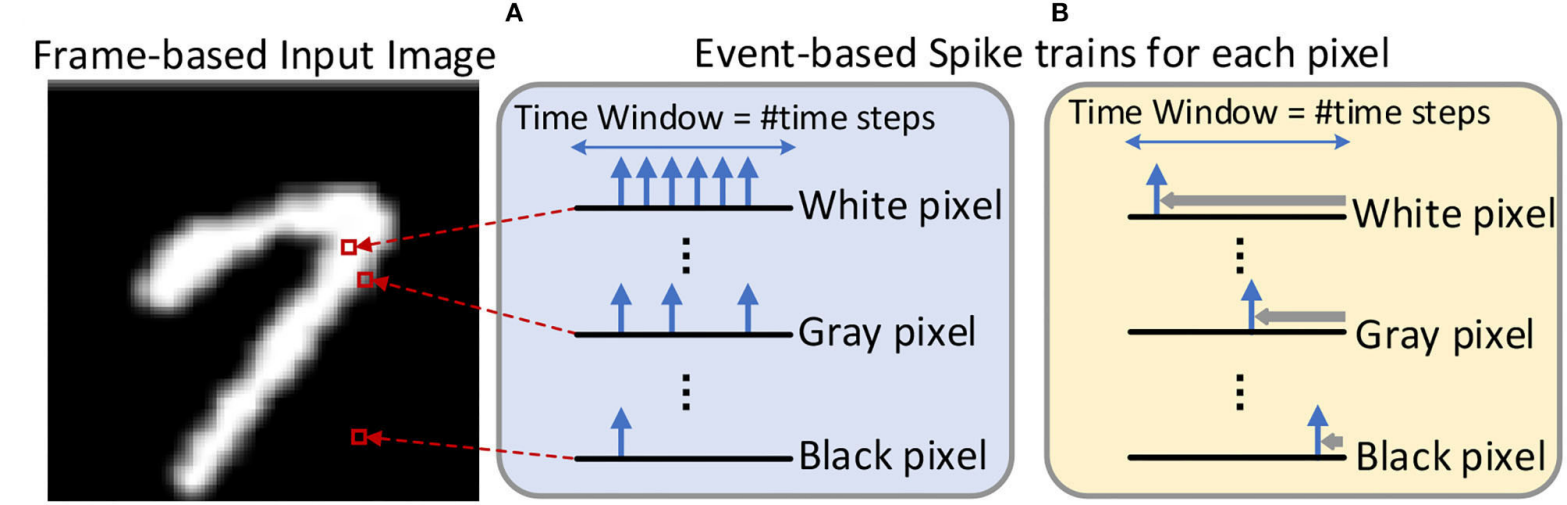
An example about the input image is converted into the input spike train by the **(A)** rate-based coding scheme (Han et al., 2020) and **(B)** Time-To-First-Spike time-based coding scheme (Rathi et al., 2020). The time window represents the length of the spike train, which is equal to the number of time steps.

During the inference, the real-valued pixel intensities of the input image are converted to the sparse spiking events over a certain time window. The time step is used to record the spike timing, and the number of time steps (also known as network latency) required is determined by the expected inference accuracy. Thus, inference in SNNs is performed on multiple feed-forward processes equal to the number of time steps, where each process requires computations based on sparse spikes. As shown in Figure 3, the two dominant coding methods are rate-based coding (Figure 3A) and time-based coding (Figure 3B) for SNNs. The rate-based coding scheme encodes the intensity of a pixel into the number of spikes, while the time-based coding scheme encodes the information as the latency to the first spike of the corresponding spike train. In the SNN with the rate-based coding scheme, massive spikes are fired to achieve accuracy comparable to the ANN, which leads to high computational costs. Therefore, the memory access and computational costs remain lower than the rate-based coding since time-based coding has only a single spike in the spike train.

#### 3.1.3. Neuron Dynamics

We use the biologically plausible Integrate-and-Fire (IF) neuron to simulate the dynamics of a spiking neuron that is driven by the input spike train via plastic synapses. The IF neuron *i* integrates the input spikes *X*_*i*_ into the current *I*(*t*) by the transmitted inter-connecting synaptic weights *w*_*i*_ of the corresponding spike and then accumulates it into the membrane potential *V*_*m*_, leading to a change in its membrane potential (*V*_*m*_). The temporal dynamics are formulated below.


(1) $$\{\begin{array}{l} \;I(t)=\sum_{i\in \{i|X_{i}(t)=1\}}w_{i}\\ \frac{\text{d}V_{m}}{\text{d}t}=I(t) \end{array}$$

Since the input values in SNN are binary spikes (i.e., “1” or “0”), the mathematical dot product operation in ANNs can be replaced by the addition in SNNs. When the accumulated membrane potential reaches a certain firing threshold, the neuron fires an output spike and then resets membrane potential. The reset mechanisms help regulate the spiking activities of the post-neurons.

### 3.2. Proposed SNN Training Methodology

#### 3.2.1. Forward Propagation

**Figure 4:**
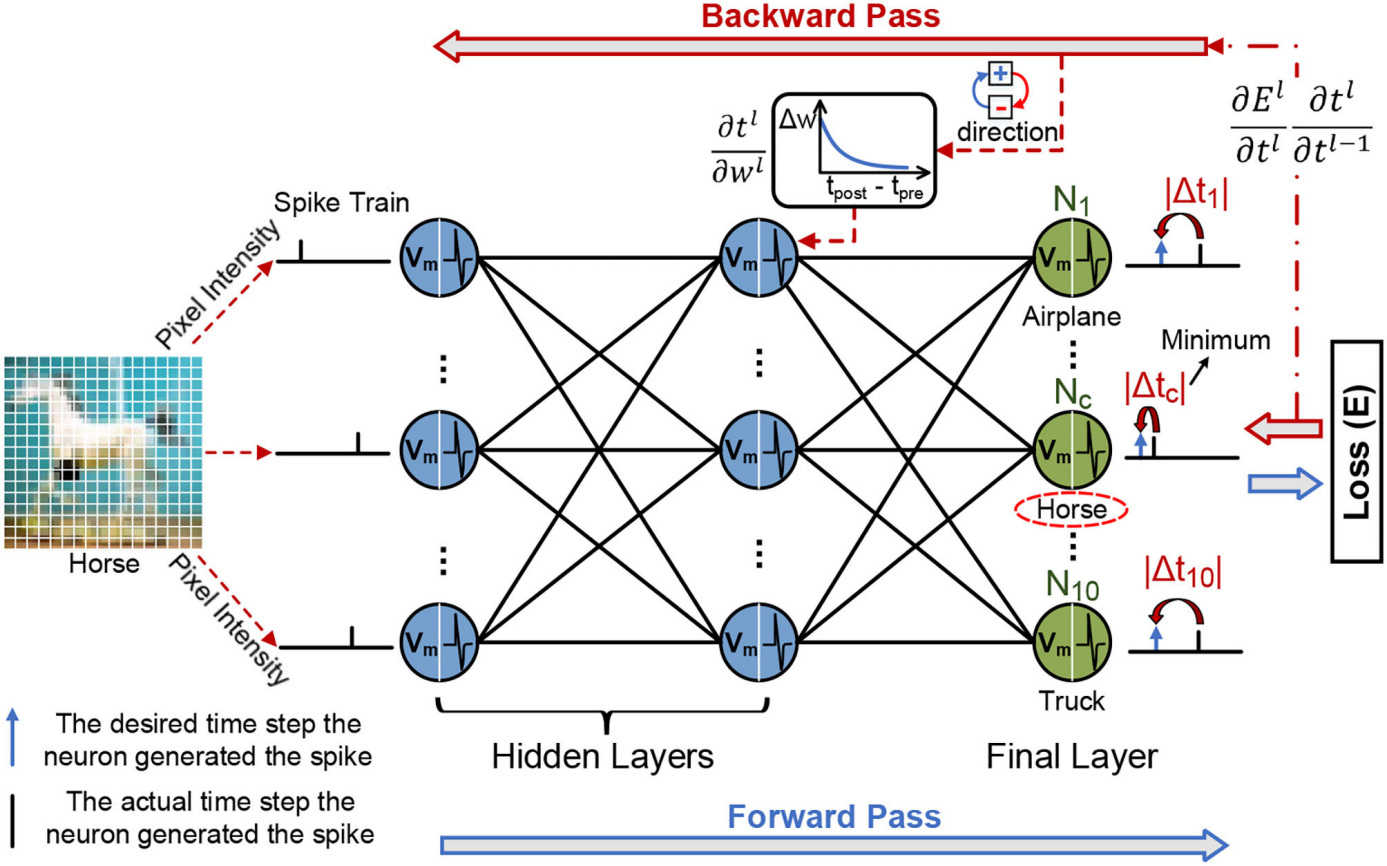
The illustration of the forward process and error backpropagation in the SSTDP method. The blue frame arrows represent forward propagation, and the red frame arrows represent backward propagation. In the forward phase, the neurons in the SNN integrate the received spikes with corresponding weights into the membrane potential and calculate the error based on the prediction results of the network. In the backward phase, the final error is backward past through the hidden layers based on the chain rule to obtain the partial derivative of the final error with respect to the time. The synaptic weights are modified with spatial (local weight updates from STDP) and temporal information (globally rectified from BP) to reduce the network error.

Figure 4 provides an overview of the SSTDP algorithm. SSTDP consists of multiple layers since the number and type of neurons (i.e., IF and LIF neurons) and layers (i.e., fully connected and convolutional layers) are not limited. Hence, one can implement SSTDP with any arbitrary number and type of hidden layers. According to Equation 1, the membrane potential *V*_*j*_(*t*) of the *j*-th neuron at time step *t*_*s*_ is computed as follows:


(2) $$V_{j}(t_{s})=V_{j}(t_{s}-1)+\sum_{i}w_{ij}X_{i}(t_{s})$$

where *X*_*i*_(*t*_*s*_) is the input spike train from the *i*-th pre-synaptic neuron, *w*_*ij*_ is the synaptic weight between the *i*-th pre-synaptic neuron and *j*-th post-synaptic neuron. The IF neuron fires a output spike with the time-based coding when its membrane potential exceeds the firing threshold θ_*j*_ (> 0):


(3) $$X_{j}(t_{s})=\{\begin{array}{ll} 1, & \text{if }V_{j}(t_{s})\leq \theta_{j}\text{ and }X_{j}(<t_{s})\neq 1\\ 0, & \text{otherwise } \end{array}$$

where *X*_*j*_(< *t*) ≠ 1 denotes to check whether the *j*-th neuron was not fired at any previous time step (< *t*_*s*_). In time-based coding, information is encoded using the spike time *t*_*s*_ of a single spike. Generally, the larger integration current is due to input spikes with larger corresponding weights. In such a scenario, a larger integration current corresponds to the possibility of the earlier fire spike, which in event-driven neuromorphic hardware can terminate the computation of the neuron earlier. Note that in the last layer (fully connected layer) in the network, the number of neurons corresponds to the number of task categories, where each neuron may fire a single spike at a different time step. After completing the forward process over the entire forward propagation time (i.e., several time-steps in the spiking domain), the category of an input image is predicted by the SNN as the category corresponding to the winner output neuron that fires the earliest spike. Since the network decision is based on the first fired spike in the last layer, earlier fired spikes carry more information in the spike train.

#### 3.2.2. Backward Propagation

To utilize backward propagation to update the weights in the neural network, we need to obtain the derivative of the loss function with respect to each weight, *i*.*e*., $\frac{\partial E}{\partial w^{l}}$, where *E* is the loss function, and *w*^*l*^ is a weight at the *l*^*th*^ layer.

In our method, the output neuron that spikes first carries the most significant signal and thus corresponds to the output label. To separate the firing time of the target neuron and others, we set a minimum gap *g* between their expected firing times. Taking the average firing time of each sample into consideration, we set the expected firing time as the following equations:


(4) $$T_{\text{mean}}=\frac{1}{n}\sum_{i=1}^{n}t_{i}^{L}$$

(5) TjL={min{tjL, Tmean−n−1ng},j=ymax{tjL, Tmean+1ng},j=y

where *n* is the number of output spikes, *y* is the correct label, $t_{j}^{L}$ is the actual firing time, and $T_{j}^{L}$ is the expected firing time. Such settings maintain the average expected firing time near the actual one to fit the firing time of each input sample and achieve better adaptation. The expected firing time of the target label is the smallest one among all output neurons with a minimum gap *g* with others to distinguish it well.

Then, the loss function can be defined as the squared error of the bias between actual firing time and expected firing time:


(6) $$E=\frac{1}{2}\sum_{j}e_{j}{\;}^{2}$$

where $e_{j}=t_{j}^{L}-T_{j}$.

Then, the gradient to the loss function can be estimated at the output layer, $\partial E/\partial t_{j}^{L}=e_{j}$, and the gradient is backward propagated to the hidden layers using the chain rule, as shown in the following equation:


(7) $$\frac{\partial E^{l}}{\partial w^{l}}=\frac{\partial E^{l}}{\partial t^{l}}\frac{\partial t^{l}}{\partial V^{l}}\frac{\partial V^{l}}{\partial w^{l}}$$

where *V*^*l*^ and *t*^*l*^ is the membrane potential and the fired spike-time of a neuron in the *l*-th layer.

As mentioned above, the term $\frac{\partial t^{l}}{\partial V^{l}}$ in Equation 7, i.e., the derivative of the post-synaptic fired spike-time with respect to its membrane potential, is not differentiable. In previous works, the derivative was estimated with various assumptions and approximations. However, estimating both ∂*t*^*l*^/∂*V*^*l*^ and ∂*V*^*l*^/∂*w*^*l*^ makes the result biased and unreliable. Therefore, our method circumvents these two non-derivable terms and merges the latter two terms into a single one:


(8) $$\frac{\partial E}{\partial w^{l}}=\frac{\partial E}{\partial t^{l}}\frac{\partial t^{l}}{\partial w^{l}}$$

According to the definition of spike-time-dependent plasticity, if the pre-synaptic spike happens before the post-synaptic one, the connection will be strengthened, making the post-synaptic spike easier to fire, and if the pre-synaptic spike occurs after the post-synaptic one, the connection is useless, and thus the weight will be reduced. Such a proposal will always make the post-synaptic spike fire earlier, neglecting the actual update direction of the post-synaptic neuron. Therefore, we only calculate the derivative between post-synaptic firing time and the weight $\partial t_{j}^{l}/\partial w_{ij}$ using STDP:


(9) $$\frac{\partial t_{j}^{l}}{\partial w_{ij}}=\{\begin{array}{ll} \epsilon_{1}(e^{-\frac{t_{post}-t_{pre}}{\tau }}-\delta )\times {\left(w_{max}-w\right)}^{\mu }, & t_{post}>t_{pre}\\ \epsilon_{2}(e^{-\frac{t_{pre}-t_{post}}{\tau }}-\delta )\times {\left(w_{max}-w\right)}^{\mu }, & t_{post}<t_{pre} \end{array}$$

where ϵ_1,2_ is the scaling factor of strengthening and restraining STDP, τ is the time constant, *t*_*pre*_ and *t*_*post*_ are the fired spike-time of a pair of pre- and post-neuron, respectively. δ represents the time interval for updating the weights in STDP, which means the spikes that occurred within this period, making a strong causal relationship between the corresponding pair of pre- and post-neuron. *w*_*max*_ and *w* are the maximum constraint on synaptic weight and the current synaptic weight, respectively. In addition, the weight update has dependence and is subject to μ. The update direction of the weight depends on both STDP and the derivative of the post-synaptic neuron, enabling STDP to learn global knowledge and lead to better performance.

To propagate the gradient to deeper layers, we further need to calculate $\partial E/\partial t_{j}^{l-1}$, which can be presented as


(10) $$\frac{\partial E}{\partial t_{j}^{l-1}}=\sum_{i}\frac{\partial E}{\partial t_{i}^{l}}\frac{\partial t_{i}^{l}}{\partial t_{j}^{l-1}}$$

The gradient of the firing time of a neuron in hidden layers is the weighted sum of all the gradients of firing time of those who receive spikes from it, and the coefficient is the derivative between them. Since the pre-synaptic spike only has an effect on the post-synaptic one when the former is earlier, we define the firing time derivative as


(11) $$\frac{\partial t_{j}^{l}}{\partial t_{i}^{l-1}}=\{\begin{array}{ll} 0, & t^{l-1}>t^{l}\\ w_{ij}^{l}, & t^{l-1}⩽t^{l}. \end{array}$$

In conclusion, the full propagation process, including both the forward and the backward pass, can be described in the following pseudo-code (Algorithm 1).

**Algorithm 1:**
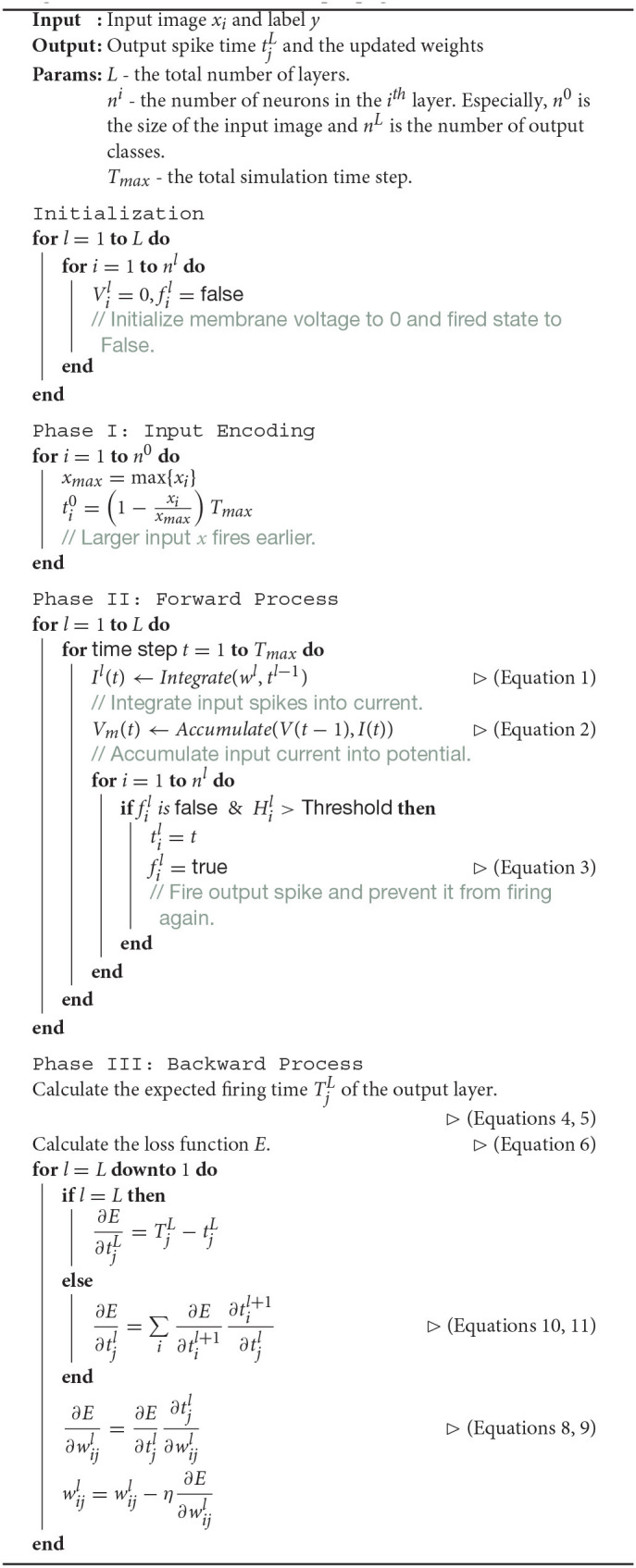
Time-based backpropagation with STDP.

## 4. Experiment

### 4.1. Experimental Setup

#### 4.1.1. Datasets

We take three visual datasets: Caltech 101 (Fei-Fei et al., 2004), MNIST (LeCun et al., 1998) and CIFAR-10 (Krizhevsky et al., 2009) for object classification tasks. Caltech 101 dataset contains 101 categories of object images. Each category has approximately 40–800 images, each of which consists of 300 × 200 pixels. Here, all the images we use are grayscaled and rescaled to 160 pixels of height. MNIST is a benchmark dataset of handwritten digits containing 60,000 training images and 10,000 testing images that have been widely used in SNN literature. Each sample of MNIST is a 28 × 28 image and contains one of the digits 0 ~ 9. CIFAR-10 is a challenging dataset for the SNN, which contains 60K RGB images in the size of 32 × 32. Following the standard practice, 50K examples are used for training and the remaining 10K for testing. The images are drawn evenly from 10 classes. There are no data augmentation tricks utilized for the MNIST dataset.

#### 4.1.2. Network Structure

To evaluate the classification performance of the proposed learning algorithm on the Caltech 101 and MNIST datasets, we consider SNN fully connected, having 784 inputs, from 300 to 700 neurons in the hidden layer, and 10 output neurons for the classification. Meanwhile, we randomly initialize the weights of hidden layers in the range [1, 10] and weights of the classification layer in the range [20, 50]. Note that the hidden layers in the network structure with spike-based classification can also be replaced by convolutional layers, which will be reported in the following experiments. To further demonstrate the effectiveness of the scheme on a large-scale dataset, we evaluated our method on CIFAR-10 using the VGG-7 network structure.

#### 4.1.3. Evaluation Metrics

To measure the estimation performance of SNNs, we employ the following metrics in terms of accuracy, speed, and energy, which are widely used in the SNN.

1) Test Accuracy. Percentage of test samples correctly classified by the SNN model.

2) Training Epoch. Passing the full training examples once through the SNN denotes an epoch. Start training models from scratch with random initialization weights, and a lower training epoch indicates the model convergence faster.

3) Time Steps. Since the real-valued inputs are encoded as the spike events over a certain time, inference in ANNS must be divided into multiple forward passes in SNNs, which are equal to the number of time steps. Thus, the number of time steps affects both the latency and the energy consumption (the number of performed operations per inference is equal to that of the sum of multiple forward processes).

4) Fired Spike Rate (FSR). The average percentage of neuron fire spikes per time step, which are used to quantify the spiking activity.


(12) $$FSR=\frac{\#\text{fired spikes}}{\#\text{total neurons}\times \#\text{time steps}}$$

A higher SR score means a larger number of spikes fired by the neuron, theoretically resulting in higher energy consumption in the neuromorphic hardware.

#### 4.1.4. Implementation Details

To valid our SSTDP algorithm, we implemented it on the PyTorch framework (Paszke et al., 2019). The weights of SNNs are initialized according to He et al. (2015). The batch size is set to 32 for the Caltech 101, MNIST, and CIFAR10 datasets to reduce memory consumption. We use the Adam optimizer (Kingma and Ba, 2014) to adjust the learning rate with the initial learning rate 5 × 10^−3^. The threshold of neurons is adjusted for different types of networks and datasets, which are typically set between 0.7 and 10. The time constant τ and constant μ in Equation 9 are set to 5 and 0.0005, respectively. For our trained SNN, we employ the IF model as the neuron model. The GPU used in training was NVIDIA RTX 2080.

### 4.2. Experimental Results

#### 4.2.1. Effect of Accuracy

First, we evaluated the test accuracy of our SSTDP method on the Caltech 101 dataset, as described in Table 1. The network performance (99.3% top-1 accuracy) of our SSTDP method outperforms other existing learning methods. Then, to further evaluate the effectiveness of our proposed SSTDP learning algorithm, as shown in Table 2, we compared the top-1 accuracy of SSTDP with recent works that directly train the SNNs from scratch based on BP on the MNIST datasets. We found that our proposed SSTDP achieves better network performance than others in terms of accuracy and latency. Specifically, SSTDP achieved the same accuracy 98.1% as the SNN trained from scratch, whereas the ANN with 400 hidden neurons and ReLU activation function achieved 98.1%. The accuracy of the SNNs trained with our SSTDP method is larger than the LeNet with 11 network depths.

**Table 1:** Test accuracy of SNNs trained with different learning methods on Caltech face/motorcycle dataset.

**Method**	**Type**	**Coding**	**Neuron model**	**Acc.** **(Top-1 %)**
R-STDP Mozafari et al., 2018	Unsupervised	Time-based	Rectified linear	98.2
SDNN Kheradpisheh et al., 2018	Unsupervised	Time-based	LIF	99.1
S4NN Kheradpisheh et al., 2020	Supervised	Time-based	IF	99.2
STiDi-BP Mirsadeghi et al., 2021	Supervised	Time-based	Linear SRM	99.2
This work	Supervised	Time-based	IF	99.3

**Table 2:** Comparsion of our work and other SNN models with direct training on the MNIST dataset.

**Method**	**Structure**	**Coding**	**Neuron model**	**Time** **steps**	**Acc.** **(Top-1 %)**
baseline (ANN)	784FC-400FC-10FC	–	ReLU	–	98.1
S4NN Kheradpisheh et al., 2020	784FC-400FC-10FC	Time-based	IF	512	97.4
STiDi-BP Mirsadeghi et al., 2021	784FC-400FC-10FC	Time-based	Linear SRM	512	97.4
Tempcoding Comsa et al., 2020	784FC-340FC-10FC	Time-based	SRM	–	97.9
STDBP Zhang et al., 2020	784FC-384FC-10FC	Time-based	Rectified linear	–	97.9
BP-STDP Tavanaei et al., 2019	784FC-1000FC-10FC	Rate-based	IF	–	96.6
TDSNN Zhang et al., 2019	LeNet	Time-based	IF	-	92.0
SNN+DT Zhou et al., 2019	784FC-Conv-Conv-10FC	Time-based	IF	–	99.33
PLIF Fang et al., 2021	784FC-Conv-Pool-10FC	Rate-based	PLIF	8	99.72
T2FSNN Park et al., 2021	VGG-16	Time-based	LIF	40	99.33
This work	784FC-300FC-10FC	Time-based	IF	16	98.1

As the number of time steps increases, more information can be represented in the spike train and can achieve higher classification accuracy. Since inference in SNNs is performed through multiple feedforward processes equal to the number of time steps (also called inference latencies), each requires computation based on sparse spikes (Deng et al., 2020; Han and Roy, 2020; Han et al., 2020; Kheradpisheh et al., 2020; Taherkhani et al., 2020). Therefore, it was noted that in other SNNs directly trained by BP, it is likely that spike signals vanish, similar to the vanishing of the gradient in ANNs. SNN thereby requires enough time steps (e.g., 512 time steps) to avoid information loss. In contrast, our method allows accuracy to be maintained even if the time steps are small (i.e., 16 time steps) and can also achieve an accuracy that equals that of the ANN because we use STDP to realize the weight gradient update and extract information. Our SSTDP has a 25.0× ~32.0× acceleration on the MNIST over others. T2FSNN (Park et al., 2021) adopt the VGG-16 as the network structure, SNN+DT (Zhou et al., 2019) and PLIF (Fang et al., 2021) use multiple convolutional layers to construct the network structure of the SNN for better accuracy.

**Figure 5:**
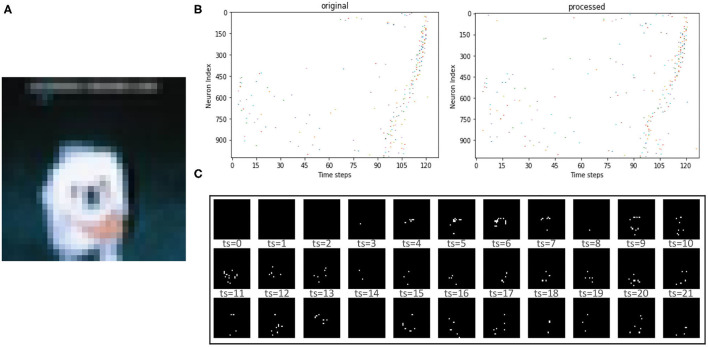
**(A)** The original image input to the SNN. **(B)** The orignial spike train input to the neurons and the processed version generated by neurons. **(C)** The reconstructed image at the first 33 time steps after time-based coding.

Table 3 lists the classification performance of all recent works on the SNN as well as our work. As can be seen on the large-scale dataset CIFAR-10 for SNNs, we also achieved an accuracy (91.31%) comparable to that of the ANN-SNN conversion network (91.46%), which out-performed other learning algorithms. Notably, previous efforts usually required 100 or even 1,000 steps to reach good accuracy (Roy et al., 2019; Wu et al., 2019b, 2021). For instance, the ANN-SNN conversion method (Sengupta et al., 2019) requires up to 2,500 time steps to maintain accuracy, the BP-based method (e.g., AFP Wu et al., 2021) requires hundreds of time steps to maintain accuracy, while we only need 16 time steps here on the CIFAR-10 dataset. Note that the inference accuracy of PLIF (Fang et al., 2021), SNN+DT (Zhou et al., 2019), SM+SR (Park and Yoon, 2021), and T2FSNN (Park et al., 2021) adopt the VGG-16 as the network structure on the CIFAR-10, while other training SNNs in Table 3 adopt the VGG-7. We found that the classification accuracy of our proposed SSTDP performs best in the time-based encoded SNN and is slightly inferior to the rate-based encoded SNN proposed in another study [1]. It is worth noting that this paper focuses on training time-based SNNs, enabling such SNNs to match or even exceed rate-based SNNs. In this way, we can ensure the prediction accuracy of SNNs and take full advantage of sparse spikes in terms of energy consumption. Figure 5 shows an example of the image from the CIFAR-10 dataset that is encoded by the time-based coding and processed by the first neuron. After processing the received spikes, every neuron, in combination with its passing synaptic weights, accumulates membrane potential. Initially, spikes in the spike trains of background pixels are fired at later time steps. After being processed by the neuron for feature extraction, the time of firing spikes is advanced. We can display the spike train as shown in Figure 5B.

**Table 3:** Test accuracy of different SNNs models on CIFAR-10.

**Method**	**Type**	**Coding**	**Neuron model**	**Time** **steps**	**Acc.** **(Top-1 %)**
DeepSNN Sengupta et al., 2019	ANN-converted	Rate-based	IF	2500	91.46
SpikeCNN Panda and Roy, 2016	Unsupervised	Rate-based	LIF	-	70.16
spike-based training Rathi et al., 2020	Supervised	Rate-based	LIF	250	90.95
direct training Wu et al., 2019b	Supervised	Rate-based	LIF	–	90.53
ASF-BP Wu et al., 2021	Supervised	Rate-based	LIF	150	90.11
Tandem learning Wu et al., 2019a	Supervised	Rate-based	IF	–	90.98
PLIF Fang et al., 2021	Supervised	Rate-based	PLIF	8	93.50
SNN+DT Zhou et al., 2019	Supervised	Time-based	IF	–	92.68
SM+SR Park and Yoon, 2021	Supervised	Time-based	IF	544	91.05
T2FSNN Park et al., 2021	Supervised	Time-based	LIF	1,280	91.36
This work	Supervised	Time-based	IF	16	91.31

#### 4.2.2. Effect of Training Epoch

**Figure 6:**
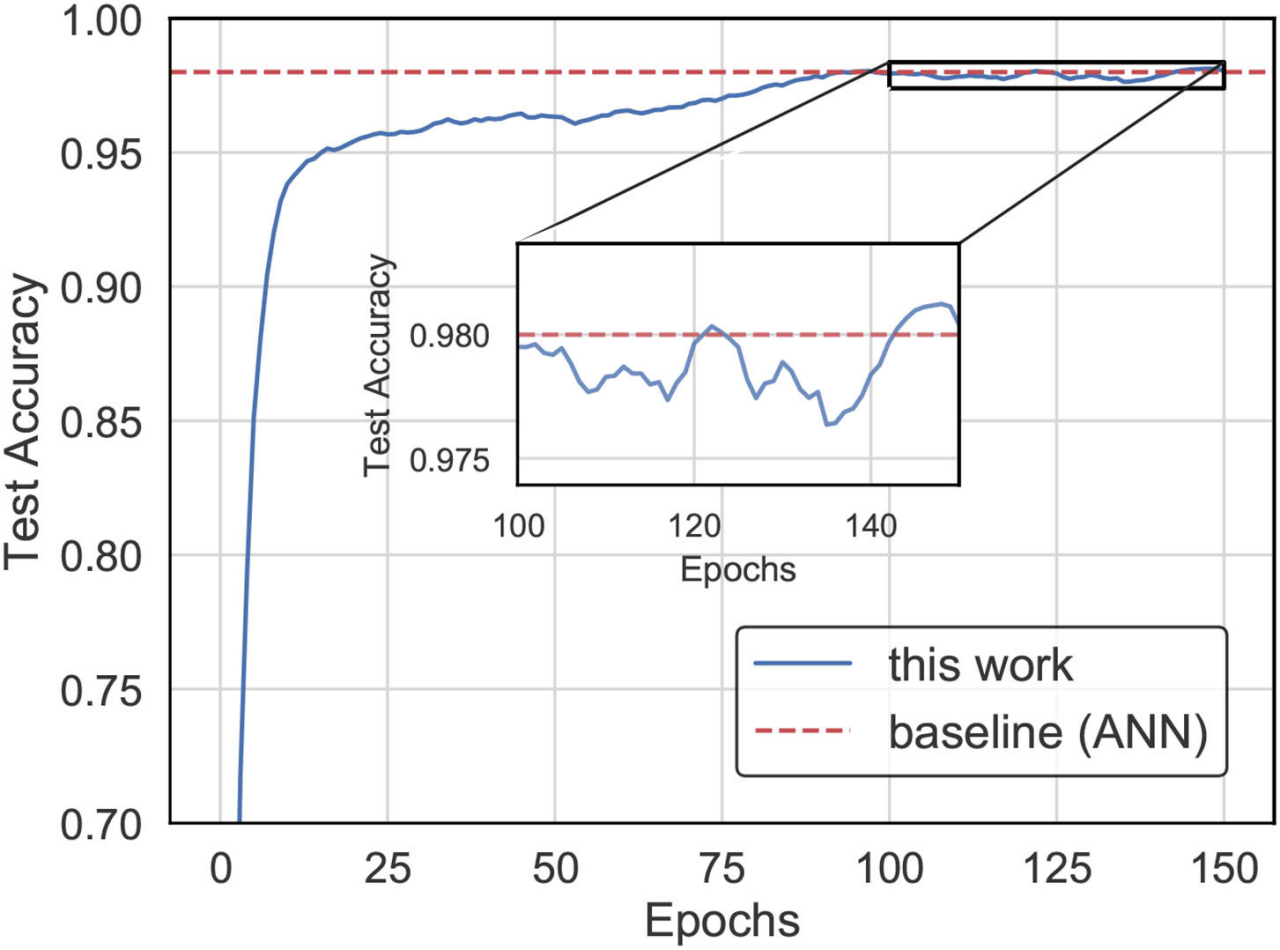
The accuracy evolution curve for training with our method on the MNIST dataset.

In this experiment, we trained the SNN for weight updating by our proposed method while using an ANN optimized by SGD (LeCun et al., 2012) for the weights as a baseline for comparison. The results in Figure 6 indicate that the SNN trained by our learning method achieves higher accuracy than the ANN with the same network structure on the MNIST dataset. It is worth mentioning that we reached the best accuracy in less than 90 epochs.

#### 4.2.3. Effect of Learning Rate Schedule

**Figure 7:**
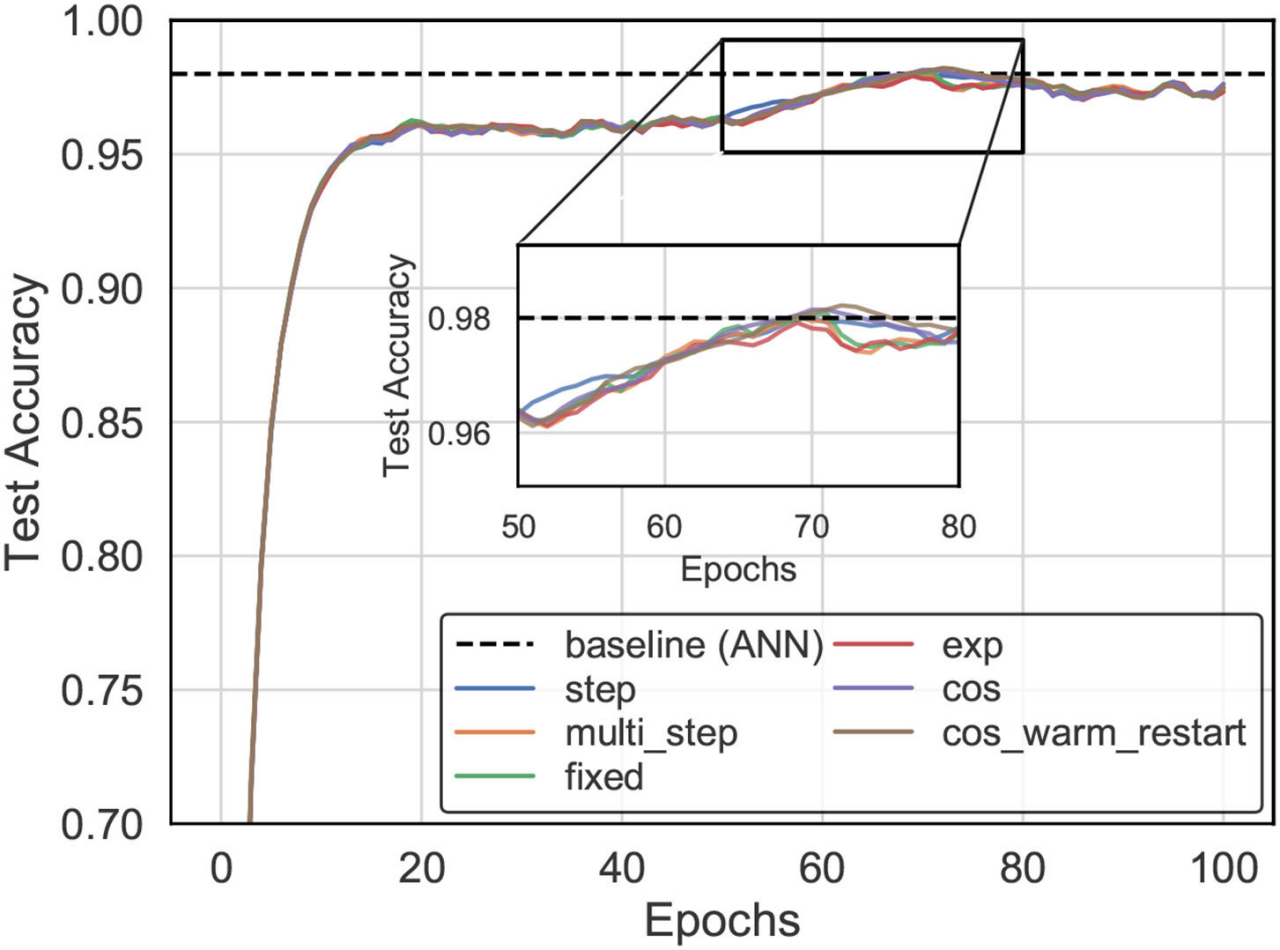
The accuracy evolution curve for training with our method on the MNIST dataset varies as the learning rate schedule.

The experiments analyzed the impact of the choice of learning rate schedule on training time and accuracy that are available in most training frameworks (Paszke et al., 2019), including fixed, exponential, step-based, multi-step-based, cosine annealing, and cosine annealing warm restart learning rate decay, as shown in Figure 7. For example, we trained an SNN model (three layers) with SSTDP for 100 epochs, using 0.003 as an initial learning rate and a step-based learning rate decay schedule (i.e., multiplied by 0.1 every 20 epochs). This model reached a top-1 accuracy of 98.1%, which is 0.14% lower than the equivalent model trained with cosine warm restarts learning rate decay.

#### 4.2.4. Effect of Time Steps

**Figure 8:**
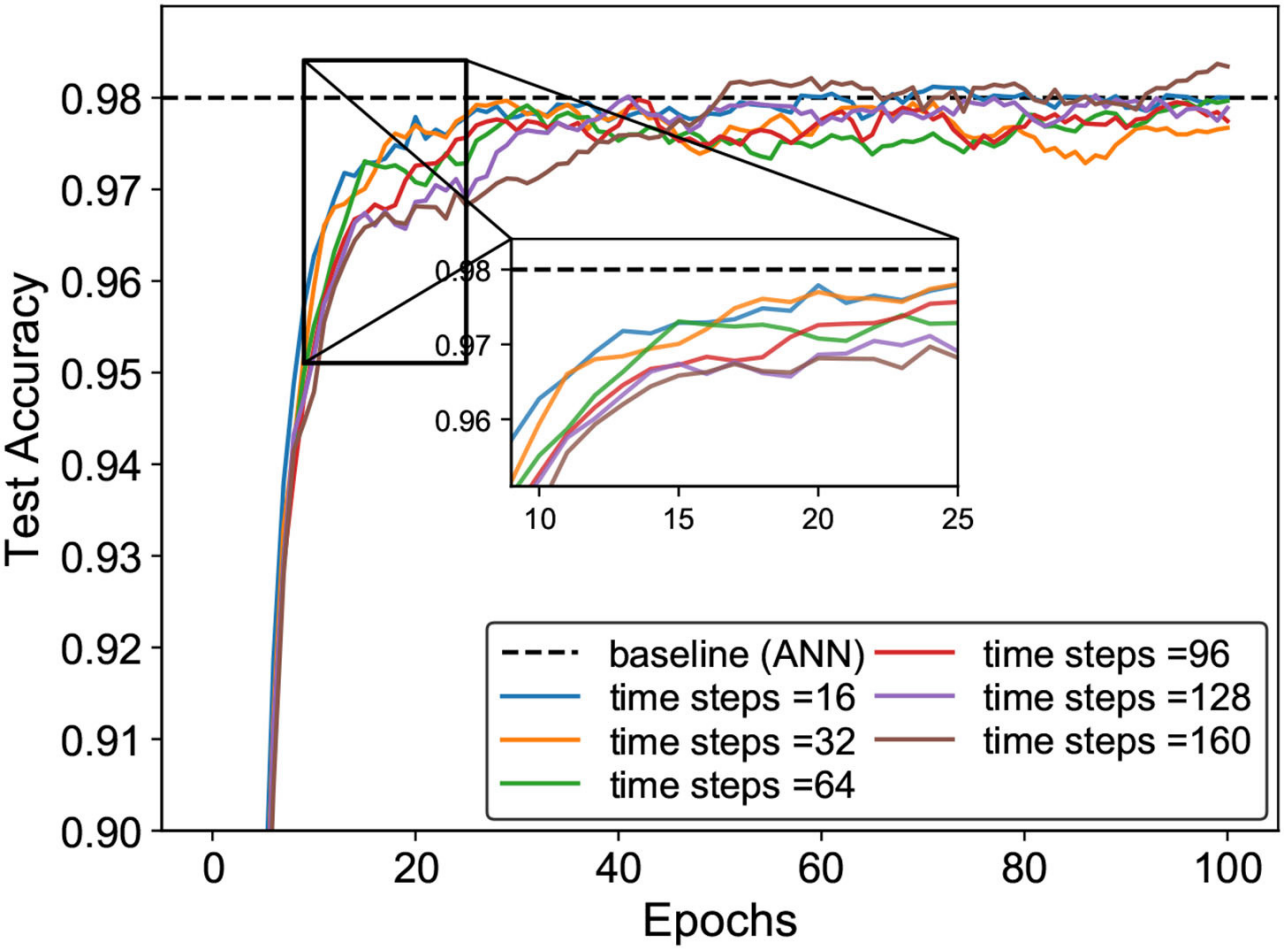
The Inference test accuracy curve of the SNN trained with SSTDP method varies as the time steps.

Figure 8 describes the test accuracy curve of the SNN trained with the SSTDP method, which varies as the time steps. We observed that the number of time steps affects the model accuracy and is also crucial for training convergence. Although the larger time steps lead to higher model accuracy, the SNN with smaller time steps converges much faster than the SNN with larger time steps. Specifically, the figure shows six curves that vary as the number of time steps increases from 16 to 160, where the model accuracy increases as the number of time steps increases. However, SNNs with smaller time steps can achieve faster convergence. This is because the SNN with smaller time steps contains more intensive information and it is easier to extract features using the local update mechanism of STDP.

#### 4.2.5. Effect of Computation Cost

**Figure 9:**
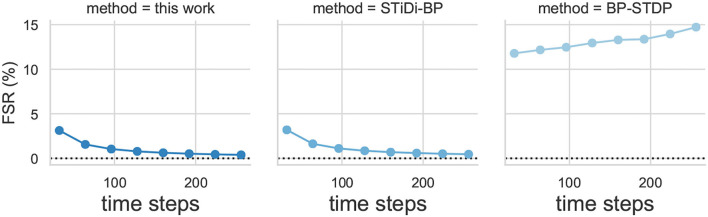
The Inference computational cost (FSR) evolution curve comparisons between SSTDP and the two baseline SNNs (STiDi-BP and BP-STDP).

**Figure 10:**
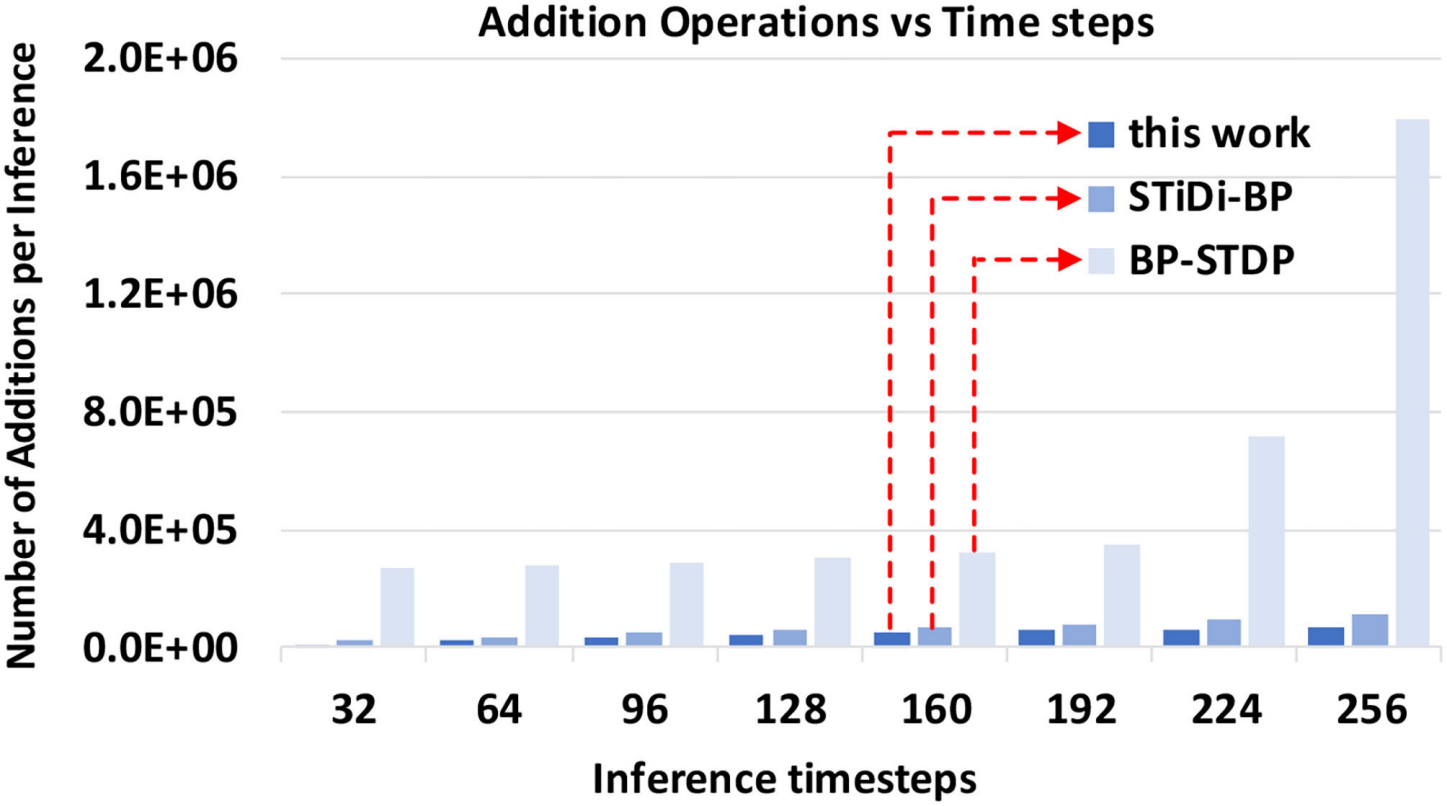
The Inference computational cost (addition operation) evolution curve comparisons between SSTDP and the two baseline SNNs (STiDi-BP and BP-STDP).

The inference computation cost of the SSTDP method, STiDi-BP and BP-STDP, are shown in Figure 9, respectively. In each figure, the x-axis is the time steps used to encode information, also considered as the SNN inference latency; the y-axis is the fired spike rate (FSR), which represents the number of operations performed theoretically for computing in SNN inference. As described in Figure 9, the proposed SSTDP method reduces the computation by orders of magnitude over the SNN with rate-based coding in Tavanaei et al. (2019) and will have an advantage over time-based SNN. Unlike our SSTDP method, other time-based approaches (Kheradpisheh et al., 2020; Mirsadeghi et al., 2021) force all neurons to fire spikes, even those that have not fired, to improve the network performance, and such an approach increases the computation effort. In addition, the network scale used in the two compared baselines is also larger than ours but with less accuracy than our method, as illustrated in the table. Specifically, our network size is only 784 × 300 × 10, while STiDi-BP and BP-STDP are 784 × 400 × 10 and 784 × 1, 000 × 10, respectively. The increase in neurons may also lead to the potential for an increase in FSR, especially for SNNs with rate-based coding. To reflect the computation more intuitively in SNNs, we also provide the number of additional operations performed in SNNs inference in Figure 10. In each figure, the x-axis indicates the SNN inference latency (i.e., time steps), and the y-axis measures the number of addition operations to compute the accumulated membrane potential in the SNN inference. We found that the proposed SSTDP reduces the number of additional operations by orders of magnitude compared to STiDi-BP and BP-STDP.

## 5. Conclusion

This paper proposes a novel supervised learning algorithm for SNNs, enabling SNNs to be implemented more efficiently by low-power neuromorphic hardware. This work establishes the bridge between the backpropagation algorithm and the STDP update mechanism, bypassing the non-differentiability part in the backward process of SNNs, using the local update mechanism of the STDP to implement it. This takes advantage of the local update property of STDP and the global signals from the BP. It enables the SNN to avoid spike signal disappearance during the execution, thus reducing the network latency. It makes the synaptic weight update receive guidance from the global signal, which guarantees the network performance. The experimental results demonstrate the advantages of our method in terms of network performance, latency, and energy consumption.

## Data Availability Statement

Publicly available datasets were analyzed in this study. This data can be found here: http://www.vision.caltech.edu/Image_Datasets/Caltech101; http://yann.lecun.com/exdb/mnist; https://www.cs.toronto.edu/~kriz/cifar.html.

## Author Contributions

FL and WZ designed the study, contributed to the source code, conducted the experiments, and evaluated the results. YC, ZW, TY, and LJ provided feedback and scientific advice throughout the process. All authors contributed to the final manuscript.

## Funding

This work was partially supported by the National Natural Science Foundation of China (grant no. 61834006 and U19B2035) and the National Key Research and Development Program of China (2018YFB1403400).

## Conflict of Interest

The authors declare that the research was conducted in the absence of any commercial or financial relationships that could be construed as a potential conflict of interest.

## Publisher's Note

All claims expressed in this article are solely those of the authors and do not necessarily represent those of their affiliated organizations, or those of the publisher, the editors and the reviewers. Any product that may be evaluated in this article, or claim that may be made by its manufacturer, is not guaranteed or endorsed by the publisher.
